# Using taTME to maintain restorative options in locally advanced rectal cancer: A technical note

**DOI:** 10.1016/j.ijscr.2020.06.015

**Published:** 2020-06-12

**Authors:** José Tomás Larach, Peadar S. Waters, Jacob J. McCormick, Alexander G. Heriot, Philip J. Smart, Satish K. Warrier

**Affiliations:** a Division of Cancer Surgery, Peter MacCallum Cancer Centre, Victorian Comprehensive Cancer Centre, Melbourne, Australia; b University of Melbourne, Melbourne, Australia; c General Surgery and Gastrointestinal Clinical Institute, Epworth Healthcare, Melbourne, Australia; d Department of Surgery, Austin Health, Melbourne, Australia; e Departamento de Cirugía Digestiva, Pontificia Universidad Católica de Chile, Santiago, Chile

**Keywords:** Transanal total mesorectal excision, taTME, Rectal cancer, Extended resection, En-bloc vaginal wall resection, R0 resection

## Abstract

**Background:**

The safe adoption of transanal total mesorectal excision (taTME) has occurred in Australasia as previously reported by the current authors. Planes beyond TME can be utilised in more advanced cases to achieve negative margins during transanal dissection.

**Methods:**

In this article we describe how taTME is used to perform an en-bloc partial vaginectomy and aid restore intestinal and vaginal continuity in a young female with a locally advanced rectal cancer and posterior vaginal wall involvement in the pre-treatment magnetic resonance imaging.

**Results:**

The transanal technique allowed the surgeons to remove a disc of vagina, ensure organ preservation and control the main R1 risk point. An R0 resection was achieved.

**Conclusion:**

This technical note highlights that in experienced hands, taTME may be safely implemented to maintain restorative options in locally advanced rectal cancer requiring resection beyond the total mesorectal excision plane.

## Highlights

• The safe adoption of transanal total mesorectal excision (taTME) has occurred in many countries worldwide.
• Planes beyond TME can be utilised in more advanced cases to achieve negative margins during transanal dissection.
• In this case, the transanal technique allowed the surgeons to ensure organ preservation and control the R1 risk point during dissection.
• An R0 resection was achieved.
• This technical note highlights that in experienced hands, taTME can be safely implemented to maintain restorative options in locally advanced rectal cancer requiring resection beyond the mesorectal plane.

## 1 Background

The management of locally advanced rectal cancer has evolved over the last two decades with a multidisciplinary approach forming the cornerstone of care [1,2]. Magnetic resonance imaging (MRI), judicious use of preoperative chemoradiation and meticulous operative planning has facilitated appropriate extended radical resections [[3], [4], [5]]. With improvements in technology, minimally invasive options are more available with the potential of organ preservation in a safe manner.

The safe adoption of transanal total mesorectal excision (taTME) has occurred in Australasia as highlighted by the our group [6], and dissection beyond the TME plane can be used in more advanced cases to perform presacral stripping, removal of endopelvic fascia or as an adjunct to remove contiguous organs [7,8].

This case depicts the application of a taTME approach to perform an en-bloc partial vaginectomy with preservation of the cervix and uterus and restore intestinal continuity in a young female. Whilst the abdominal intent was to perform a robotic resection, despite an unexpected abdominal conversion the utility of the transanal technique is highlighted in this case.

This work has been reported in line with the SCARE criteria [9] and approved by the institutional ethics committee.

### 1.1 Case description

**Fig. 1:**
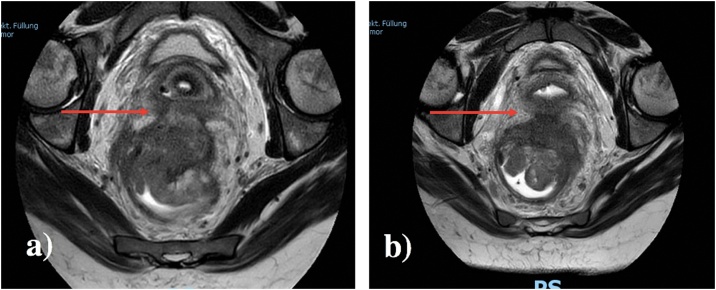
Pretreatment MRI: a) Large rectal tumour likely invading cervix (red arrow), b) rectal tumour invading posterior vaginal wall (red arrow).

A 31-year-old, otherwise well female, with a body mass index of 26.2 kg/m^2^, presented with a locally advanced, mismatch proficient rectal cancer. She had had 12 months of symptoms, including altered bowel habit, rectal bleeding and increasing obstructive-type symptoms. Her initial staging with MRI and chest-abdomen-pelvis computed tomography (CT) showed a bulky mucinous rectal cancer with invasion of the posterior vaginal wall and possibly the cervix (Fig. 1). Locoregional lymph nodes were enlarged, including pre-sacral and external iliac nodes. There was no evidence of further distant disease.

The patient was re-staged 4 weeks after completion of chemoradiotherapy with a fluorodeoxyglucose (FDG)-positron emission tomography (PET)-CT and MRI. An exam under anaesthesia was also performed to assess vaginal involvement. These results confirmed an anteriorly based tumour, 6 cm from the anal verge with posterior vaginal wall invasion and potential cervical invasion, enlarged right pelvic side-wall lymph nodes and no further distant disease.

A discussion at the exenterative multidisciplinary team meeting suggested utilising a robotic approach from above and taTME approach from below as a combined two-team approach.

## 2 Materials and methods

### 2.1 Transabdominal phase

The operation was carried out seven weeks after completion of chemoradiation. The operating team was set up for a synchronous two teams approach (Cecil approach) (surgeons SW/JM). The intent was to perform a robotic abdominal component including complete splenic flexure mobilisation being performed at the time of initial transanal set up. Unfortunately, at the outset of the robotic operating component a very proximal inferior mesenteric artery (IMA) injury occurred that required immediate but controlled conversion (only second reactive conversion in over 250 robotic cases). IMA bleeding was controlled using a gauze and a laparoscopic grasper to occlude the defect through the assistant port whilst the robot was de-docked and a midline laparotomy was performed. The abdominal surgery was continued as an open procedure. This included proximal suture of the IMA with interrupted 4-0 prolene, controlling the bleeding. The IMA was divided and suture ligated with 0 Vicryl and afterwards the left colon and sigmoid colon were mobilised in a lateral to medial fashion with identification and protection of left ureter, nerves and gonadals. Medially the peritoneum at the level of the sacral promontory was incised and continued cranially to the root of the IMA. Further dissection was carried out cranially to the duodenal-jejunal ligament allowing proximal ligation and division of the inferior mesenteric vein. Complete splenic flexure mobilisation was performed following access into the lesser sac via division of supra-colic omentum with continuation laterally to meet the lateral dissection plane along Toldt´s fascia. The colon was transected proximal to the level of the IMA with 2-0 Vicryl ties to the mesenteric vessels and marginal artery and division of the colon performed with a GIA™ 80 mm linear stapler. The abdominal contents were packed superiorly to allow commencement of the abdominopelvic dissection.

The abdominopelvic approach included right ureterolysis, posterior TME plane dissection followed by dissection of the superior pelvic peritoneum in the pouch of Douglas to facilitate partial mobilisation of the recto-vaginal septum anteriorly and the entry into the posterior vagina. Despite preoperative concerns about posterior cervical invasion, this was clearly not the case intraoperatively where planes were easily dissected and non-involved up to the cranial portion of the posterior vagina.

This component was then ceased until the taTME team developed the anterior plane between rectum and vagina in order to control the potential R1 resection margin. The transanal component defined the distal vaginal entry point.

A complete right lateral pelvic side-wall dissection was performed commencing at the level of the common iliac bifurcation dissecting inferiorly into obturator fossa, preserving the obturator nerve and vessels. Medialisation of the right ureter was necessary. The nodal harvest of obturator fossa and the internal iliac system was performed. A full description of our approach to a side-wall has been provided previously [10].

### 2.2 Transanal phase

**Fig. 2:**
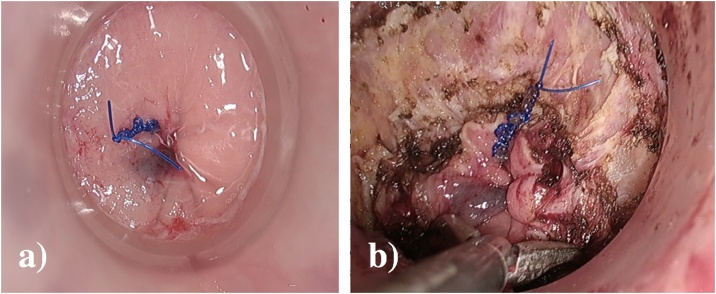
a) Transanal prolene purstring with 1-0 prolene, b) full-thickness rectotomy along.

**Fig. 3:**
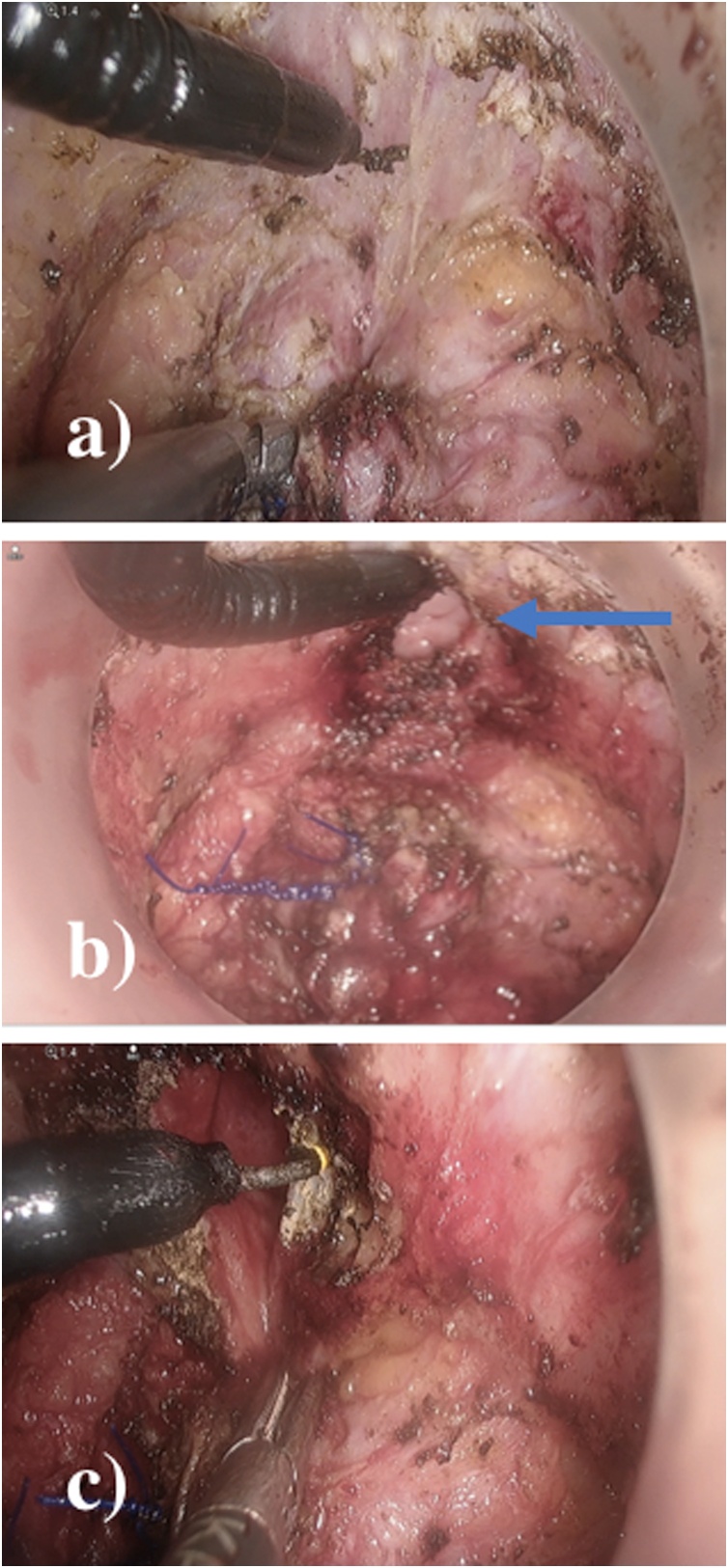
a) Anterior dissection through rectovaginal septum, b) posterior vaginal wall breakthrough at the inferior vaginal margin (blue arrow), and c) dissection of vaginal stalks laterally, transanally, with hook diathermy.

**Fig. 4:**
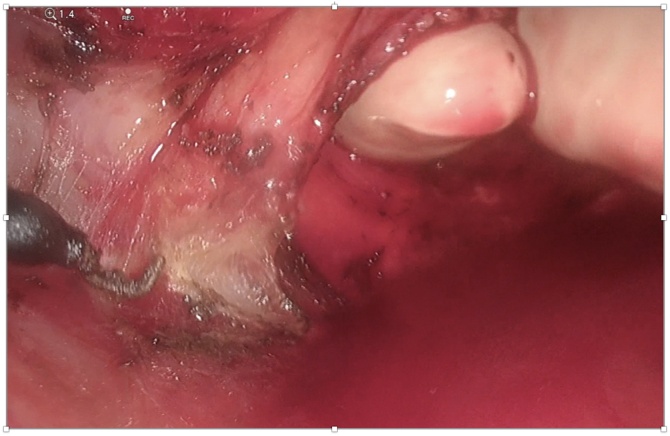
Transabdominal assistance for transanal dissection of posterior mesorectal plane.

The taTME setup was performed synchronously. A Lone star retractor (Cooper Surgical) was applied allowing eversion of the anoderm. The GelPOINT® Path (Applied Medical) was placed in the anal canal and thorough washing with cetrimide performed. A gauze was placed in the anus, and 1-0 prolene suture with a 26 mm rounded needle was used to create the pursestring in a standard clockwise fashion ensuring equidistant sutures from the port cuff (Fig. 2a). Three ports were utilised for the dissection, with initial 0-degree Olympus camera at low pressure used to facilitate a complete rectotomy. Using the AirSeal® System (CONMED) the pressure was increased (5 mmHg–12 mmHg) to create pneumo-insufflation in the limited space and facilitate smoke evacuation [11]. Circumferential markings were made with diathermy and the dissection was continued performing a full-thickness rectotomy (Fig. 2b). Following identification of the endopelvic fascia the extrafascial TME plane was identified. The anterior plane was selected based on continuous bimanual palpation but included dissection anterior to the anterior condensation of fascia (Fig. 3a). Control of the anterior margin was aided by continuous communication of both primary surgeons and with the use of a bimanual exam allowing a posterior full thickness vaginectomy 1 cm inferiorly to the invasive component (Fig. 3b, c). Following this the lateral stalks of the vagina were taken by the transanal approach and the proximal margin divided by the abdominal surgeon from above under direct visualisation of the transanal surgeon. This allowed for control of the potential R1 risk point. Dissection was completed in a coordinated effort by both teams transabdominally and transanally (Fig. 4). The transanal approach also facilitated right pelvic side-wall clearance distally including obturator fossa clearance by taking endopelvic fascia on that side to access the caudal portion of the obturator fossa.

The specimen was extracted transabdominally with primary repair of the posterior vaginal wall with interrupted full thickness 2-0 Vicryl sutures. A colonic pouch was fashioned, and a single stapled anastomosis created with an EEA™ 33 mm circular stapler (Medtronic).

## 3 Results

Total operative time was 220 min and blood loss approximately 400 mL. The patient had an uneventful recovery and was discharged on day six post-operatively.

### 3.1 Pathology

**Fig. 5:**
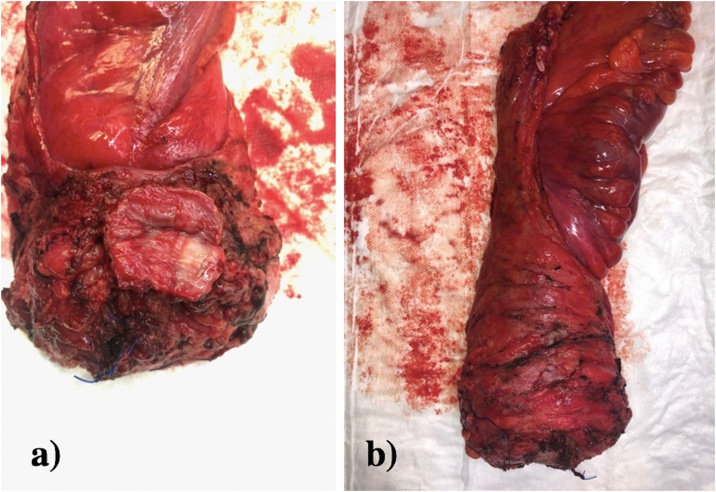
Surgical specimen: a) Anterior aspect of specimen showing an en bloc resection of a posterior vaginal wall disc and b) right posterolateral vision of specimen showing intact mesorectum.

On macroscopic inspection of the specimen inspection the vaginal disc looked complete with clear margins and the mesorectum was intact throughout (Fig. 5). Histopathology revealed pathological downstaging with microscopic residual primary adenocarcinoma in the mid rectum, through the full thickness of the anterior rectal wall, but not into the vagina (ypT3). Tumor regression grade was a Ryan grade 1. Circumferential and distal resection margins were well clear from tumour. All mesorectal nodes were negative and a right pelvic side-wall lymph node was positive for adenocarcinoma (1/2). TNM staging was ypT3N0M1a.

### 3.2 Follow-up

The patient completed adjuvant chemotherapy (CAPEOX), and had her stoma closed 10 months after surgery. After 12 months of follow-up she has no local or distant recurrence.

## 4 Discussion

This case highlights the merits of adopting a combined abdominal and taTME approach for surgical resection of complex anterior rectal tumours to achieve an R0 resection. A partial posterior vaginectomy has been performed with preservation of the uterus utilising this approach and hence allowed for colonic and vaginal reconstruction. To our knowledge, this is the first reported case in the literature.

Planes beyond the TME dissection can be utilised in select cases to allow for multivisceral resection [7,8]. Here, the transanal technique allowed the surgeons to preserve the sphincters, remove a disc of vagina, therefore preserving the distal vaginal wall and control the main R1 risk point. The R1 risk point indicates the points or series of points where the surgical margin is at greatest risk of microscopic disease involvement and is a term we have coined previously [7]. In this case, this point is best seen transanally.

The case again highlights the importance of having specialist colorectal surgeons involved in the utilisation and adoption of new technology. Both SW/JM are exenterative minimally invasive surgeons and SW/AH are credentialled robotic and taTME proctors. The technique represents an incremental alteration as we move forward with more organ preserving approaches for complex rectal cancers. The personalised decision to preserve the uterus was made due to the patients young age and possible future fertility as the fertility field grows in the future, despite having had chemoradiotherapy. The intraoperative findings supported such an approach.

The authors recommend that such approaches occur in institutions that are high volume rectal cancer centres with appropriate specialised experience.

## 5 Conclusion

TaTME can be safely implemented to maintain restorative options in locally advanced rectal cancer requiring resection beyond the mesorectal plane.

## Declaration of Competing Interest

The authors declare that they have no conflict of interest.

## Funding

Epworth Medical Foundation.

## Ethical approval

This study is exempt from ethnical approval in our institution.

## Consent

Informed consent was obtained from all patients included in the study and a copy is available for the Editor-in-chief by request.

## Authors contribution

Satish Warrier, Jacob McCormick, Alexander Heriot, Peadar Waters and Jose T. Larach were the surgeons involved in this case. Satish Warrier and Jacob McCormick were the primary surgeons. Jose T. Larach, Peadar Waters, Philip Smart and Satish Warrier wrote the article. Al authors reviewed the final version of the manuscript.

## Registration of research studies

NA.

## Guarantor

Satish K. Warrier.

## Provenance and peer review

Not commissioned, externally peer-reviewed.
